# Does Printing Orientation Matter? In-Vitro Fracture Strength of Temporary Fixed Dental Prostheses after a 1-Year Simulation in the Artificial Mouth

**DOI:** 10.3390/ma14020259

**Published:** 2021-01-07

**Authors:** Julian Nold, Christian Wesemann, Laura Rieg, Lara Binder, Siegbert Witkowski, Benedikt Christopher Spies, Ralf Joachim Kohal

**Affiliations:** Medical Center–University of Freiburg, Center for Dental Medicine, Department of Prosthetic Dentistry, Faculty of Medicine, University of Freiburg, Hugstetter Str. 55, 79106 Freiburg, Germany; christian.wesemann@uniklinik-freiburg.de (C.W.); noemi.rieg@uniklinik-freiburg.de (L.R.); lara.binder@icloud.com (L.B.); siegbert.witkowski@uniklinik-freiburg.de (S.W.); benedikt.spies@uniklinik-freiburg.de (B.C.S.); ralf.kohal@uniklinik-freiburg.de (R.J.K.)

**Keywords:** additive manufacturing, fracture strength, printing orientation, anisotropy, stereolithography (SLA), fixed dental prostheses

## Abstract

Computer-aided design and computer-aided manufacturing (CAD–CAM) enable subtractive or additive fabrication of temporary fixed dental prostheses (FDPs). The present in-vitro study aimed to compare the fracture resistance of both milled and additive manufactured three-unit FDPs and bar-shaped, ISO-conform specimens. Polymethylmethacrylate was used for subtractive manufacturing and a light-curing resin for additive manufacturing. Three (bars) and four (FDPs) different printing orientations were evaluated. All bars (n = 32) were subjected to a three-point bending test after 24 h of water storage. Half of the 80 FDPs were dynamically loaded (250,000 cycles, 98 N) with simultaneous hydrothermal cycling. Non-aged (n = 40) and surviving FDPs (n = 11) were subjected to static loading until fracture. Regarding the bar-shaped specimens, the milled group showed the highest flexural strength (114 ± 10 MPa, *p* = 0.001), followed by the vertically printed group (97 ± 10 MPa, *p* < 0.007). Subtractive manufactured FDPs revealed the highest fracture strength (1060 ± 89 N) with all specimens surviving dynamic loading. During artificial aging, 29 of 32 printed specimens failed. The present findings indicate that both printing orientation and aging affect the strength of additive manufactured specimens. The used resin and settings cannot be recommended for additive manufacturing of long-term temporary three-unit FDPs.

## 1. Introduction

The fabrication of fixed dental prostheses (FDPs) is rarely achieved in one session. Therefore, a temporary solution is needed to bridge the duration between preparation and cementation of the final FDP. A temporary restoration protects the prepared tooth from chemical, thermal, and physical irritations and restores chewing function, esthetics, and phonetics, as well as fixing the tooth position [1,2]. Long-term temporaries can also be used to test a new bite position [3]. While short-term temporaries can be manufactured chairside, long-term temporaries are fabricated in the dental laboratory based on conventional or digital impressions.

After conventional impression-taking, the tooth morphology can be restored using a wax-up to create a negative mold for subsequent fabrication of a temporary restoration made of chemically or light-curing resins. As an alternative, a digital workflow including computer-aided design (CAD) and computer-aided manufacturing (CAM) is feasible. This allows the manufacturing of the temporary and final FDP based on the identical data set [4].

In the case of CAD–CAM manufacturing, most of the material is discarded when the temporary restoration is milled, and reuse of the resulting waste is not possible at present [5]. In contrast, during additive manufacturing, only the volume of the temporary restoration and supporting structures are cured, making this procedure more resource-efficient.

The most widespread additive technology in dentistry is vat photopolymerization, whereby a liquid photopolymer in a vat is selectively cured by light-activated polymerization [6]. The two most established methods are stereolithography (SLA) and digital light processing (DLP). In the first case, the polymerization is performed by a directed UV-laser point; in the second case, a whole layer is simultaneously polymerized by a UV-light mask [7]. After printing, the parts have to be cleaned from excess monomer in isopropanol and then post-polymerized with UV-light.

The mechanical properties of additively manufactured parts are not only influenced by the material but also by the manufacturing process. The post-processing protocol is of crucial relevance. Post-curing time, the radiant power and wavelength of the UV-curing unit, as well as the temperature can influence the material properties [8,9]. Likewise, these are influenced by the printing orientation leading to an anisotropic behavior of the parts [10]. As reasons for this, the interlayer bond [11] and technology-based differences in the local polymerization process [12] are discussed. For geometric reference bodies, a vertical printing orientation when perpendicular loads are applied shows the highest load capacity [10]. However, little is known about to what extent this can be transferred to complex morphologies such as FDPs. In addition, the materials must withstand intraorally dynamic loads, a wet environment, and thermal stress.

Therefore, this study aimed to investigate first, the flexural strengths of subtractive manufactured versus additive manufactured reference bodies of different printing orientations according to ISO standards. The flexural strength is defined as the maximal stress reached during a three point flexural test, measured in MPa. Second, it compared the fracture strength of milled versus additive manufactured three-unit FDPs of different printing orientations by means of static loading. The fracture strength is defined as the exerted force at the moment of fracture during a static loading test, measured in N. Third, it investigated the impact of dynamical loading and thermal stress of a chewing simulator on fracture resistance. The null hypothesis assumed that neither the manufacturing method nor the printing orientation influenced the flexural and fracture strength.

## 2. Materials and Methods

### 2.1. Fabrication and Static Loading of Bar-Shaped Specimens

**Figure 1:**
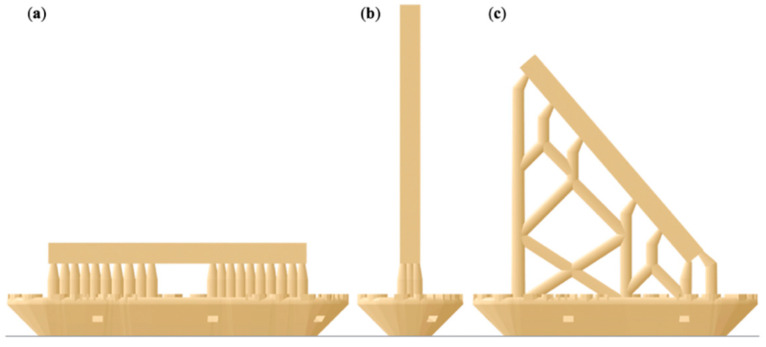
Illustration of the bar-shaped specimens in an (**a**) horizontal, (**b**) vertical, and (**c**) diagonal printing orientation including support structures and rafts.

A bar-shaped specimen with the dimensions of 25 × 2 × 2 mm was designed in a CAD software program (Tinkercad, Autodesk, San Rafael, CA, USA) and exported as a standard tessellation language (STL) file. Acting as our control group, eight bar specimens were subtractive manufactured out of the commonly used polymethylmethacrylate (PMMA) blanks for provisional restorations (inCoris, Dentsply Sirona, Charlotte, NC, USA). This was achieved using a five-axis milling machine (MC X5, Dentsply Sirona) quipped with the recommended PMMA bur set (0.5, 1.0, and 2.5 mm bur, Sirona) and the highest quality setting (inLab Software, Sirona). For additive manufacturing of 24 bar specimens, the design was digitally orientated in a vertical, diagonal, and horizontal position on the print platform (PreForm Software, Formlabs, Boston, MA, USA). Eight specimens of each orientation were printed using an acrylic resin (Denture Teeth, Formlabs) and an SLA printer (Form2, Formlabs) using a layer height of 50 µm (Figure 1).

Postprocessing of the printed samples included a 10 min wash in 99% isopropanol (Form Wash, Formlabs) followed by UV-curing for 60 min at 60 °C (Form Cure, Formlabs). After removing the supports, this post-curing process was repeated twice with the samples being submerged in vaseline. This was done to prevent an oxygen inhibition layer and, therefore, a layer of uncured resin that would negatively affect biocompatibility when used in the patient’s mouth. The objects were rotated by 180° in-between the latter two post-curing steps.

After finishing, they were placed in distilled water for 24 h at 37 °C and measured in height and width with a digital caliper (accuracy of 0.01 µm; DealMux, Guangzhou, China). The flexural strength of both materials and the influence of the printing orientation were determined in a three-point bending test in accordance with ISO 4049 [13] as well as ISO 10477 [14]. The three-point bending test until fracture was performed using a universal testing machine (Z010/TN2S, ZwickRoell, Ulm, Germany) with a loading span of 20 mm and a crosshead speed of 1 mm/min. The maximum flexural strength, *σ*, in megapascal (MPa) was calculated with the following Equation (1):

(1) $$\sigma =\frac{3FL}{2bd^{2}}$$

*F* is the maximum load in Newton, *l* is the distance between the supports in millimeters (20 mm), *w* is the width in millimeters (2 mm), and *h* is the height in millimeters (2 mm).

### 2.2. Fabrication and Static and Dynamic Loading of FDPs

#### 2.2.1. Preparation of Specimens

**Figure 2:**
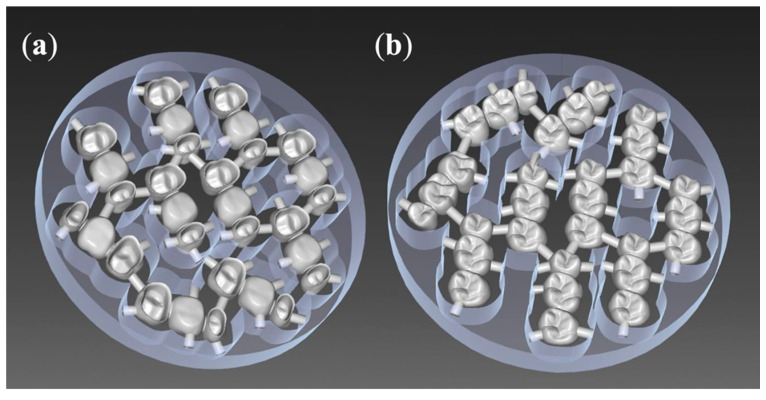
Ten fixed dental prostheses (FDPs) nested into one olymethylmethacrylate(PMMA) blank (inCoris, Dentsply Sirona): (**a**) bottom view, (**b**) top view.

For the standardized fabrication of three-unit FDPs, the upper right first molar (tooth 16, according to the FDI scheme) was removed from a phantom model (KaVo Dental, Biberach, Germany), and the upper right second premolar (15) and second molar (17) were prepared with a circular chamfer of 0.8 mm and an occlusal reduction of 1.5 mm. This situation was digitized with a model scanner (inEos X5, inLab software, Dentsply Sirona), and a three-unit FDP was designed following the recommended settings of the inLab software for the fabrication of long-term temporaries out of inCoris PMMA blanks. This resulted in connector sizes of 15.05 mm^2^ mesial and 14.07 mm^2^ distal of the pontic 16 (Figure 2).

**Figure 3:**
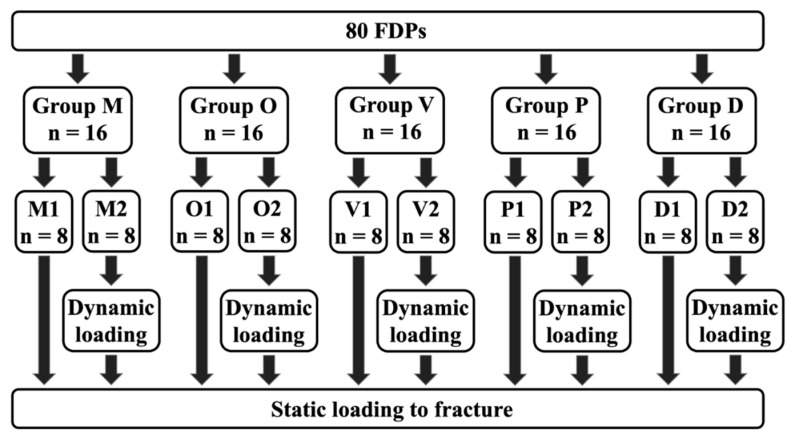
Groups: (M) milled, (O) occlusal, (V) vertical, (P) palatal, (D) diagonal, (1) non-aged, (2) aged.

As our control group for both aged and non-aged specimens, 16 milled FPDs (M1 n = 8, M2 n = 8) were manufactured, similar to the bar-shaped specimens, out of PMMA blanks (inCoris, MC X5 milling machine, Dentsply Sirona) (Figure 3).

**Figure 4:**
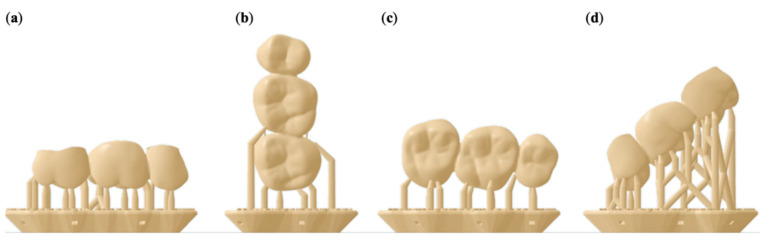
Illustration of the FDP specimens in an (**a**) occlusal, (**b**) vertical, (**c**) palatal, and (**d**) diagonal printing orientation including support structures and rafts.

For additive manufacturing of the FDPs (n = 64), four printing orientations were used (Figure 4). Group occlusal (O): Occlusal surface pointing down towards the print platform. Group vertical (V): The distal side of the FDP is facing the print platform. Group palatal (P): The palatal side of the FDP is facing the print platform. Group diagonal (D): Positioning at a 45° angle with the mesial side facing the print platform.

The supports were only placed on the outside of the FDP; no internal supports were used. All FDPs of groups O, V, P, and D were printed and post-processed similar to the bar-shaped specimens.

A total of 80 FDPs were produced, consisting of five groups of 16 specimens each.

#### 2.2.2. Preparation of Object Holders

**Figure 5:**
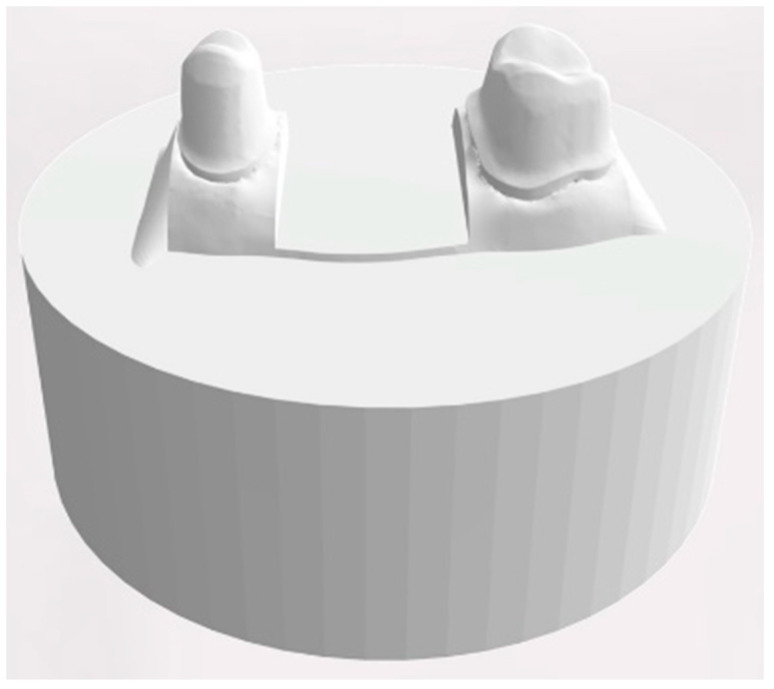
Design of the object holder with two prepared teeth.

For designing a standardized object holder, the scan of the dental model was reduced to the area from stump 15 to 17 and exported. A cylindric bottom part with a diameter of 4 cm and a height of 1.5 cm was generated using a CAD software program (Tinkercad, Autodesk) and merged with the reduced dental model into a single STL file (MeshMixer, Autodesk) (Figure 5).

Based on this design, 80 object holders were printed in acrylic resin (Rigid, Formlabs) on the SLA printer (Form2, Formlabs) using a layer height of 50 µm. After the print was completed the object holders were cleaned for 15 min using 99% isopropanol (Form Wash, Formlabs) and UV-cured for 30 min at 60 °C (Form Cure, Formlabs).

All FDPs were cemented to their object holders with zinc oxide-based cement for temporary cementation (TempBond NE, Kerr, Bioggio, Switzerland) following the recommendations of the manufacturers at a controlled pressure of 80 N.

### 2.3. Dynamic Loading with Simultaneous Hydrothermal Cycling

The 16 samples of all five groups were subdivided into (1) eight specimens remaining as manufactured and (2) eight that were artificially loaded and aged in a computer-controlled dual-axis chewing simulator (CS4.8, Willytec, Munich, Germany) by means of dynamic loading and hydrothermal cycling.

Dynamic loading consisted of a vertical load of 98 N applied at the center of the occlusal surface by means of a three-point support (mesio buccal, mesio palatal, and distobuccal cusp) of 16 with a subsequent lateral side shift of 0.5 mm under load. To simulate one year of clinical loading, 250,000 cycles were chosen [15]. Hydrothermal cycling included an exposure to water set at 5 °C for 30 s, a drain time of 10 s, followed by an exposure of 30 s to water set at 55 °C. The status of the FDPs was visually controlled twice per day.

### 2.4. Static Loading

All bridges, the non-loaded as well as those that survived the dynamic loading procedure in the artificial chewing simulator, were loaded until fracture at the previously described three-point contact of 16 at a speed of 10 mm/min using the universal testing machine (Z010/TN2S, ZwickRoell). The maximum load (Fmax) was recorded.

### 2.5. Statistical Analysis

Normal distribution (Kolmogorov–Smirnov test) and variance homogeneity (Levene-test) of the data were verified. Afterward, the flexural strength (MPa) of the bar-shaped specimens and the maximum load (Fmax) of the FDPs were analyzed by one-way ANOVA with post-hoc Bonferroni pairwise comparisons. The analysis was performed with a statistical software program (SPSS Statistics, v22.0, IBM, Armonk, NY, USA). The significance level was set at α < 0.05.

## 3. Results

### 3.1. Static Loading of Bar-Shaped Specimens

The group of milled bars showed the highest mean fracture strength with 113.6 ± 9.8 MPa (Table 1). All printed groups showed significantly lower values (*p* = 0.001), with the vertically printed bars showing significantly higher flexural strength compared to the diagonally and horizontally printed groups (*p* < 0.007).

### 3.2. Dynamic Loading with Simultaneous Hydrothermal Cycling of FDPs

**Figure 6:**
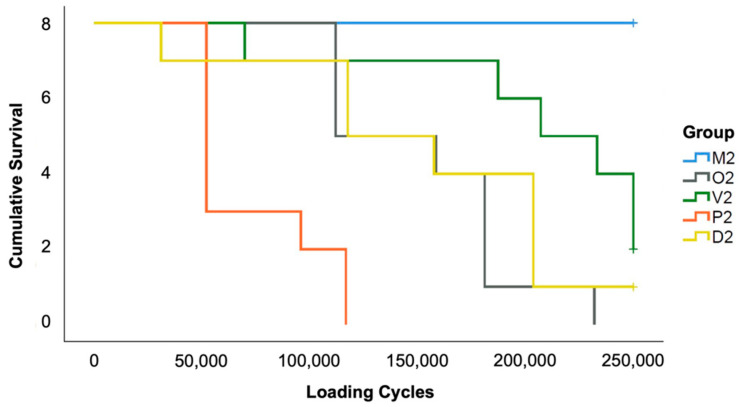
Kaplan–Meier survival rates of the FDPs during thermodynamic loading.

While all milled specimens sustained the artificial loading (group M2), the additive manufactured specimens failed more often. In group P2 and O2, all specimens fractured. In group D2 only one and in group V2 two out of eight specimens survived the aging procedure (Figure 6).

### 3.3. Static Loading of FDPs

One-way ANOVA of the untreated samples revealed significant differences between the groups (*p* = 0.001). The mean fracture load of group M1 (1060.1 ± 88.9 N) showed significantly higher values compared with all additive manufactured specimens, except group D1 (*p* = 0.311). Among the printed specimens, D1 showed the highest load capacity (931.7 ± 151.3 N) and P1 the lowest (727.6 ± 107.3 N, *p* > 0.011).

**Figure 7:**
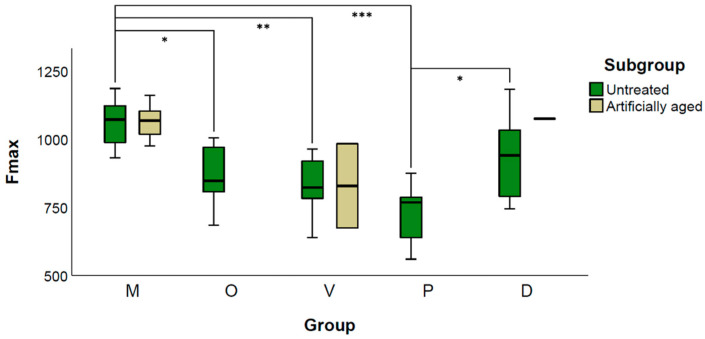
Grouped Boxplot of Fmax (N) of untreated and artificial loaded FDPs. Additive manufactured specimens revealed a total failure during artificial aging in group O and P, one survivor in D and two in V represented by the upper and lower limits of the box. * indicates *p* < 0.05; ** indicates *p* < 0.01, *** indicates *p* < 0.001.

When comparing M1 with M2 (1064.3 ± 61.3 N), no significant difference was found (*p* = 0.931). The two surviving FDPs from group V2 showed a fracture strength of 983.5 N and 674.3 N, whereas the surviving specimen of group D2 fractured at an applied load of 1075.2 N (Figure 7).

### 3.4. Fracture Analysis

**Figure 8:**
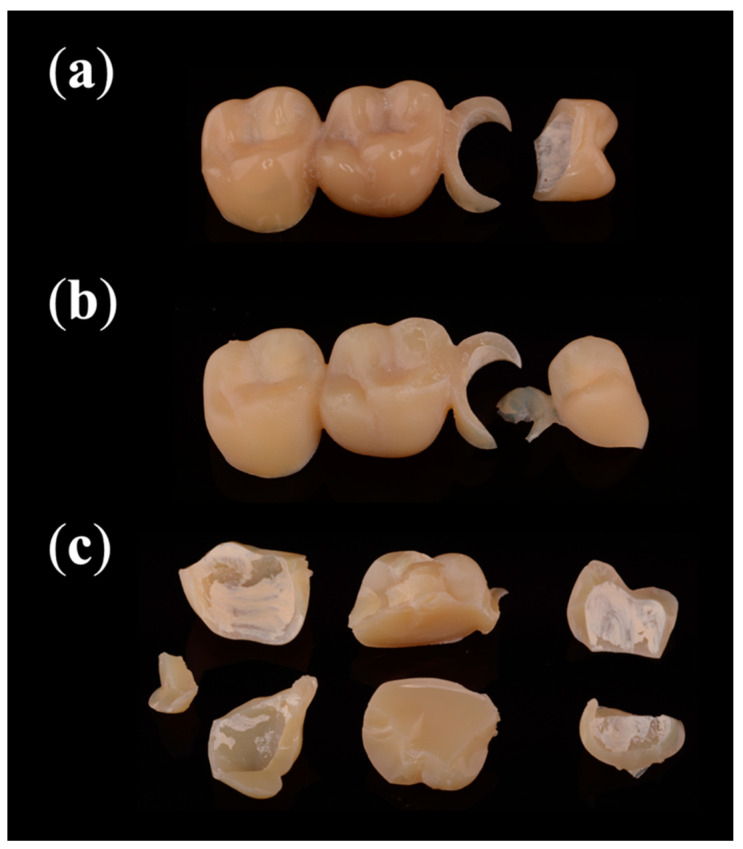
Most common fracture patterns: (**a**) statically loaded subtractive manufactured FDPs and (**b**) dynamically loaded additive manufactured FDPs fractured into two pieces, while (**c**) statically loaded additive manufactured FDPs fractured into multiple pieces.

All 16 subtractive manufactured FDPs fractured into two parts when statically loaded (Figure 8). Nine samples fractured between crown 15 and its connector (Table 2); the other seven showed a fracture affecting 15, the connector, and 16. During artificial aging, 29 out of 32 additive manufactured FDPs fractured. Of those, 93% fractured into two pieces. The prevalent failure patterns were a fractured connector of 15 (83%), a fractured pontic (76%), and a fractured premolar crown (72%). All statically loaded specimens fractured into multiple pieces.

## 4. Discussion

In this in vitro study, the effect of printing orientation of additive manufactured temporary FDPs on fracture strength was compared to subtractive manufactured samples. The fracture strength was statically determined according to available ISO standards using bar-shaped specimens as well as statically and dynamically by means of artificial loaded FDPs. As a result, the fracture resistance was significantly affected by the manufacturing technique, the printing orientation, and in the case of the additive manufactured FPDs, the applied loading procedure. Therefore, the null hypothesis had to be rejected. Subtractive manufactured specimens showed the highest loading capacity. A vertical printing orientation revealed the highest values for bar-shaped specimens, whereas a diagonal printing orientation showed the highest values for FDPs. Dynamic loading with simultaneous hydrothermal cycling did not affect the fracture strength of the subtractive manufactured FDPs, while most of the additive manufactured specimens failed during this procedure.

The combined testing of bar-shaped specimens and FDPs was intended to evaluate whether the results of standardized ISO-conform specimens can be transferred to complex organic morphologies. Since the materials are exposed to complex loads and temperature fluctuations in the oral cavity, the FDPs were additionally dynamically loaded and hydrothermally cycled in the chewing simulator. A vertical load of 98 N representing applied forces during mastication was applied [16]. This is consistent with comparable studies [17]. Additionally, each loading cycle included a lateral movement by means of a 0.5 mm side shift with the applied load of the antagonist. This simulates complex masticatory motions and represents higher stress compared to solely vertically applied forces [18]. Furthermore, since some materials are known to be less fatigue-resistant when exposed to an aqueous environment [19,20], hydrothermal cycling in water changing the temperature from 5 to 55 °C every 30 s was included during dynamic loading. In previous studies, it was discussed that rigid sample holders led to a reduction in the fracture load and may not reflect the biological conditions [8,21]. For this reason, customized sample holders were made of resin instead of prefabricated steel mounts to mimic the dampening effect of the periodontal fiber apparatus between the alveolar bone and teeth.

The choice of cementation material can affect the fracture strength of crowns [22]. In this study, eugenol-free temporary cement was used for cementation. Nakamura et al. [23] showed that their adhesively cemented zirconia crowns achieved higher fracture loads compared to those cemented conventionally. In addition, Stawarczyk et al. [22] showed that leucite reinforced glass-ceramic crowns achieved significantly higher fracture loads using an adhesive cement acting as a stress breaker, but no such effect was found for resin-based crowns. Whether adhesive cementation would have resulted in higher fracture loading capacities in this study remains unknown. However, adhesive cementation of temporary FDPs is currently not to be considered a clinical standard procedure.

All bar specimens achieved significantly higher flexural strength than the 50 MPa required by the ISO 10477:2020 standard [14], but only the subtractive manufactured bar specimens achieved more than the required 100 MPa of the ISO 14049:2019 standard [13]. The vertical printing orientation showed the highest mean flexural strength of all printed bar specimens (96.9 MPa) with an increase of 16% compared to the other additive manufactured bars. This is in accordance with Unkovskiy et al. [10] who showed that the specimens with layer orientation parallel to the axial load achieved superior flexural strength. The horizontal and diagonal printing orientation showed similar results. Meanwhile, new materials containing ceramic fillers are available on the market which may reveal improved strength.

Regarding the FDPs, a high failure rate of the additive manufactured groups under dynamic loading with simultaneous thermocycling occurred. Therefore, the additive manufactured groups with and without artificial aging could not be compared to each other due to the resulting low sample size. For the subtractive manufactured FDPs, artificial aging did not end in a statistically significant difference regarding fracture strength. Static loading of the FDPs revealed that the subtractive manufactured group showed a significantly higher fracture strength compared to all additive manufactured specimen, except those printed in a diagonal orientation. In accordance with Park et al. [11], the group with the palatal printing orientation of the FDPs exhibited the lowest fracture strength. The diagonally printed FDPs showed the highest load capacity, but the differences to the vertical and occlusal printing orientations are minor and might not be of clinical relevance.

The data obtained in the present investigation for FDPs are not directly comparable to other studies. Many factors such as the span length and design of the FDPs influence the load capacity. Reymus et al. compared different parameters that affect the fracture strength of CAD–CAM fabricated temporary FDPs [8]. The load capacities in their investigation ranged from 777 to 1050 N, depending on the used resin. Milled samples showed comparable results to the printed ones (881 N), but the control group using chairside autopolymerizing bis-acryl methacrylate exhibited significantly lower fracture strengths (552 N). This is in agreement with the values of Park et al., who showed comparable fracture strength values for FDPs after milling, SLA, and DLP printing, but reduced results for chairside autopolymerizing [11].

The fracture analysis revealed that the milled FDPs fractured under static load into two pieces, while the additive manufactured ones fractured into multiple pieces. The pontic was prone to fracture longitudinally under static loading. It could be explained by the force exerted through the round shape of the indenter resulting in a transversal force and spreading the buccal and palatal cusps apart. This effect is to be expected to be less pronounced during the dynamic loading with a force of only 98 N, explaining the different fracture behaviors.

The anisotropy of additive materials caused by the printing orientation is known [24]. The demonstrated increased load capacity of the vertically printed bar-shaped specimens is in line with previous investigations [10,24]. For the two most common technologies, SLA and DLP, this can be explained by two different phenomena. In the case of SLA, the laser speed is slower in the marginal areas and leads to a higher degree of polymerization than in central areas [12]. When vertically printed, the ratio is improved in favor of the marginal areas [25]. Using DLP, the UV-light is projected over an assembly of micro-mirrors, resulting in a simultaneously polymerized layer consisting of a multitude of voxels. In the vertical direction, the voxels are polymerized without gaps forming columns layer by layer. In the lateral direction, however, the voxels are separated from each other by thin interstitial areas showing a reduced degree of polymerization. These areas correspond to the boundaries of each micro-mirror, which may represent a potential weakness [12]. When vertically oriented, long columns are present, whereas flat orientation results in many short columns with correspondingly numerous interstitial areas. If the anisotropy of the parts is not explained by the interlayer bonding, but by varying laser speed between marginal and central regions, it is comprehensible that complex morphologies such as FDPs can show a different optimal printing orientation compared to bar-shaped specimens. In this study, a diagonal printing orientation showed the best results for FDPs. This is consistent with the results of Park et al., who showed increased loading capacity when FDPs were diagonally printed [11]. However, the effect of printing orientation for SLA and DLP is overlaid by other factors. Layer height [24], post-processing parameters such as wavelength [8], and radiant power [9], as well as water absorption [26], have a greater influence on the mechanical properties.

The described weakening effect through water absorption is in line with our results of the artificially aged groups. The non-aged specimens withstood at least 559 N and up to 1183 N during static loading. However, almost all failed during dynamic loading with 98 N and simultaneous thermocycling. Väyrynen et al. used geometric reference bodies to investigate the influence of printing orientation and water storage on fracture strength [26]. Printing orientation showed a minor influence with slightly better values for vertical and diagonal orientation. In contrast, water storage of 14 days reduced the load capacity of the specimens by about 50%. Berli et al. demonstrated that two out of three investigated additively manufactured resins absorbed significantly more water than milled polymers [27]. For both materials, the fracture strength after water storage was reduced by about one-third. This is in accordance with our results of the additive manufactured and artificially aged FDPs that showed a comparable fracture strength to the non-aged groups after being no longer subjected to an aqueous environment. The third printable resin in their study, which showed only low water uptake, exhibited almost the same strength in the water-saturated as in the dry state. The water uptake is reversible [27], which explains why the surviving FDPs after dynamic loading and drying showed comparable loading capacity to the initial state.

The resin used in this study has no ceramic fillers. Newer resins that incorporate ceramic particles might show improved mechanical properties. Artificial saliva would have allowed for a closer representation of the intraoral situation, but because of the risk of mineral depositions, only the use of distilled water is allowed in the chewing simulator.

In summary, the printing orientation affected the flexural strength of standardized bars and the fracture strength of the FDPs. Assuming varying laser speeds as the reason for those results, the morphology of the printed object is of decisive importance. General recommendations for the printing orientation can, therefore, not be given. The effect of water storage and artificial aging of the additive manufactured specimens has to be regarded as detrimental. This fatiguing resulted in failure significantly below the initial load capacity of the additive manufactured specimens. Accordingly, when using materials for restorations in patients, not only ISO conform flexural tests are required, but also the validation of fatigue after simulated long-term loading and water immersion under clinically realistic conditions is highly important.

## 5. Conclusions

The subtractive manufactured bars and FDPs showed the highest strength in all experiments. The strength of the additive manufactured specimens was affected by the printing orientation. While vertical printing was superior for the bar-shaped specimens in terms of flexural strength, diagonal printing orientation showed the highest fracture strength for the FDPs. According to our results, the palatal printing orientation should be avoided. Additive manufacturing of the utilized material for the FDPs showed acceptable fracture strength in the dry state, but dynamic loading with simultaneous hydrothermal cycling decreased the strength in a clinically relevant way.

## Figures and Tables

**Table 1:** Mean and standard deviation (SD) of the flexural strength of the subtractive and additive manufactured bars in megapascals (MPa).

Technology	Group	N	Mean	SD
subtractive	milled	8	113.6 * MPa	9.8 MPa
additive	horizontal	8	82.8 ** MPa	4.2 MPa
additive	vertical	8	96.9 *** MPa	9.9 MPa
additive	diagonal	8	83.4 * MPa	3.6 MPa

Groups with the same asterisks count did not differ significantly from each other.

**Table 2:** Areas of failures during dynamic and static loading of FDPs.

Technology	Loading	N	Crown 15	Connector 1	Pontic 16	Connector 2	Crown 17
subtractive	dynamic	0	0	0	0	0	0
	static	16	16	16	7	0	0
additive	dynamic	32	21	24	22	8	7
	static	35	34	35	34	34	31

## Data Availability

Data is contained within the article.
